# Performance indicators in speed climbing: insights from the literature supplemented by a video analysis and expert interviews

**DOI:** 10.3389/fspor.2023.1304403

**Published:** 2023-12-22

**Authors:** Somayeh Askari Hosseini, Peter Wolf

**Affiliations:** ^1^ Faculty of Physical Education and Sports Sciences, Kharazmi University, Tehran, Iran; ^2^ Sensory-Motor Systems Lab, Department of Health Sciences and Technology, ETH Zurich, Zurich, Switzerland

**Keywords:** performance analysis, anthropometrics, physical fitness, biomechanics, physiology, strategy and technique

## Abstract

Due to the goal of mastering a standardised route with relatively large handholds as quickly as possible, the weighting of performance-determining factors might be different in speed climbing in comparison to lead climbing or bouldering. The objective of this paper was to identify physical and tactical factors for peak performance in speed climbing. Therefore, not only existing literature was reviewed but also a video analysis of the final round of speed climbing at the Olympic games and interviews with experts were done. Out of two hundred and one articles initially found by searching in Medline, Elsevier and Google Scholar databases, 38 were ultimately considered. Generally, an increased lower limb power, a small body mass index, an improved anaerobic glycogen system, and a high fluency in movements were identified as characteristics for high-speed climbing performance. Based on video analysis of Olympic games, coordinated motions, correct foot movements and shorter reaction time could take a novice speed climber to an elite level. Furthermore, male climbers should avoid pairing hands on holds. Considering the increasing popularity, the continued improvement of the record time in this discipline, and the currently limited amount of relevant literature should stimulate future studies on performance-determining variables, assessments, and training methods to maintain the attractiveness of speed climbing.

## 1. Introduction

Speed climbing is a discipline of sport climbing in which an athlete attempts to get as quickly as possible to the top timing pad. Thus, in speed climbing, it is all about time, which makes the rankings easy for non-experts to follow, which in turn makes speed climbing attractive to the media. For this reason, the International Olympic Committee initially considered allowing speed climbing to become an Olympic event as a separate discipline. When the combination of speed climbing, bouldering, and lead climbing was finally announced, the interest in speed climbing has skyrocketed. More and more climbing gyms permanently provide at least one speed climbing route. In addition, the number of members of climbing associations and sports clubs participating in speed climbing competitions increases continuously, for instance in New Zealand by almost 20% each year since the announcement that speed climbing has been included in the Olympic Games (1). An examination of the results of the women's world speed climbing championships revealed that the performances of the top athletes became increasingly similar in recent years (2), which indicates an increase in the overall level of women specialising in speed climbing.

In 1998, a speed climbing competition was held for the first time. At that time, athletes competed on two different routes to achieve the lowest possible total time. The routes changed from competition to competition, which prevented personal best times or world records. This was only made possible by the standardisation of the route and holds. The current 15 m parallel track was introduced in 2007 on the occasion of the World Championships in Aviles. The essential characteristics of the wall, which also applied to the 2020 Tokyo Olympics, have been defined by the International Federation of Sport Climbing (IFSC) as follows: The wall consists of two routes running parallel to each other (at a distance of about 1 m). Each route consists of 20 square panels with a side length of 1.5 m, arranged two by ten on top of each other. The wall must overhang regularly 5°. The surface has to be light grey, covered by resin quartz. The two types of holds, 20 big handholds and 11 small footholds, are formed after an official master hold provided by the IFSC. Competition holds are equally coloured in bright red, but at the last Olympics there were three black handholds on each course to measure the split times (holds provided by Luxov, France). The usual mechanical-electronic timing system consists of a starting pad, recording when the athlete lifts their feet off the ground, and a stop device, which must be hit at the top of the wall. The timing system should record with an accuracy of 1/1000 of a second. Also, the timing system shall indicate a false start, which occurs when an athlete leaves the starting pad before the last beep of the start sequence has sounded for 0.1 s. Belaying is performed via automatic belay systems at the top anchor. It must be a certified system if a world record is to be recognised (3).

Successful climbing requires the interaction of physical aspects (specific physical fitness, anthropometric characteristics, coordination skills, and other biomechanical and physiological variables), mental aspects (personality traits, temperament), and tactical aspects (climbing strategy and technique). To improve climbing performance, all aspects have to be trained in an appropriate manner (4). The percentage importance of the aspects varies in different sports, but in lead and bouldering they are about the same (5). In speed climbing, cognitive aspects are less important during the run, as the movement sequence is hardly adapted because the route is known. As for intermediate climbers lead climbing is more mentally demanding than climbing when belayed via a top rope (6, 7), the mental challenge for non-elite speed climbers during training could be less.

Speed climbing is still a relatively young sport and the factors that determine success have not yet been researched very much. Who can be successful in speed climbing and why? What are typical tactics of the best speed climbers and which deficits should beginners work on? How are performance-determining factors weighted in speed climbing compared to lead climbing or bouldering? These questions are addressed in this paper by evaluating the current literature on speed climbing to compile existing knowledge on the performance-determining factors in speed climbing. In addition, a video analysis of the last Olympic Games was carried out and interviews with experienced coaches and a former World Champion who was a World record holder were conducted to provide a sophisticated basis for training activities and to point out possible future research directions. Nowadays, video analysis is a simple tool for recognising, for instance, movement sequences, errors, and strategies. Complemented by the experts' perspective, we wanted to gain further insights into the factors that influence performance to provide crucial knowledge about speed climbing for athletes and trainers.

## 2. Method

This work tried to gather information about the performance of speed climbing through a literature review, a video analysis of the Olympic Games, and expert interviews.

### 2.1. Literature review

Medline (PubMed), Elsevier, and Google Scholar databases were searched for published primary manuscripts and reviews. The following keywords were used: “performance”, “success”, “speed climbing”, “climbing”, the Boolean operator “AND” combined with “indoor climbing” or “velocity “or “sport climbing”, and additionally “NOT” linked with “animals” or “ladder “or “stairs” or “injury “or” psychology”. Abstracts and full texts from the earliest available record until May 2022 were considered, and reference lists of primary manuscripts and reviews were manually evaluated to retain additional relevant studies.

Descriptive, experimental, correlational, and case studies as well as non-peer-reviewed papers, and reports of speech about speed climbing, each available in either Persian, Russian, or English were eligible. Primary inclusion criteria of studies were: (A) The population of interest had to be speed climbing athletes. (B) Focus of the study had to be on physiology, anthropometry, specific physical fitness, or biomechanics. Studies considering speed as a factor of physical fitness, peace of movement in other disciplines of climbing and studies reporting speed climbing as one discipline of sport climbing were excluded. Articles that had been written in narrative syntheses, psychology and mental areas, pathology domain and engineering sciences were rejected. Further, all manuscripts were excluded concerning equipment or speed climbing of objects or animals (Table 1).

**Table 1:** List of inclusion and exclusion criteria.

Criteria	Inclusion	Exclusion
Language	English, Russian, Persian	All other languages
Population	Speed climbers	Lead and boulder climbers, animals, objects
Study type	Descriptive, experimental, correlational, case studies, non-peer-reviewed papers, reports of speech	Narrative syntheses
Scope	Physiology, anthropometry, specific physical fitness, biomechanics	Psychology, mental area, pathology domain, engineering sciences, speed as a factor of physical fitness or part of movement

### 2.2. Video analysis of speed climbing at the Olympic games in Tokyo

Video analysis is an important method in analysing the performance of speed climbing without specialized equipment (8). Based on video analyses of competitions, the training of tactical, technical and physical aspects of speed climbing can be improved (9). To gather information about tactics of elite speed climbers, a video stream of the finals in climbing at the Olympic Games was recorded and both the women's and men's attempts in speed climbing were examined by the first author. Each attempt was viewed at least five times at half playback speed regarding the following criteria:

• Use of hands (which holds were used and did the hands grasp alternately and simultaneously?)
• Use of feet (sequence and placement on holds or directly on the wall)
• Position/orientation of the body on the wall
• Movement at start
• False starts and movement errors such as placing a foot incorrectly on a hold or the wall surface or impractical grasping the holds with the hands resulting in lower speed or even stopping on the track or falling.

### 2.3. Expert interview

All training methods are based on personal experience without being substantiated by scientific studies. As hardly any guideline exists on technique, tactics, and training methods in speed climbing—and since it was important to us to give practical tips, semi-structured interviews were conducted with three experienced speed climbing coaches and trainers of the Iranian national team and a champion of the world (Reza Alipoor) to complement our literature review. The interview included six basic questions:

• How can male and female climbers achieve better times? Please explain separately.
• What are the most prevalent mistakes made by novice and elite speed climbers, both female and male?
• Do you think speed climbing athletes can achieve higher skill levels easier, i.e. from novice to elite, than athletes of other climbing disciplines? Why?
• Do you prefer doing more high dynos with less gripping than short ones catching more holds?
• Is laddering (i.e., alternate grasping) a better strategy than grasping holds with both hands simultaneously (pairing)?
• Any comments about current practical training methods and exercises for speed climbing athletes?

## 3. Result

**Figure 1:**
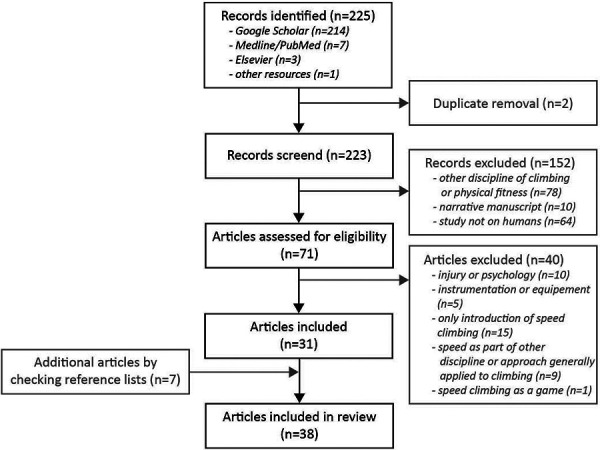
Overview o f the systematic literature search: PRISMA flow chart.

A total of 225 articles were found through the database search. After the first author (S. Askari) removed two duplicates, the abstracts of the remaining articles were read and reviewed for exclusion criteria. As a result, 152 articles were then excluded. After the remaining 71 articles were read in full, another 40 articles were excluded. A review of the reference lists resulted in the inclusion of seven additional articles, ultimately resulting in 38 eligible articles (see Figure 1).

To allow for a structured discussion of the literature, articles were assigned to one of the following four areas: anthropometrics, physiology, motor control, and biomechanical metrics characterising movement quality (in Supplementary Table S1, providing an overview on all studies included, the same structure is considered).

A total of 38 articles were included. One particular data set was exploited in four articles, another for two articles. Notably, seven articles were published in non-indexed peer-reviewed journals.

The results of the video analysis are presented in a specific section in the discussion. Input of the experts from the interviews complement the discussion of articles whenever appropriate (whereby the answers from the interviews were also consolidated in Supplementary Table S2).

## 4. Discussion

### 4.1. Anthropometrics

Elite speed climbers were reported to have a higher body weight (67.2 kg) than recreational climbers (65.7 kg) (10). However, their body fat was found to be lower (13.4% against 17.2%), so their higher body weight results from their muscle mass. Sit-ups in 30 s, standing board jump tests, and hand dynamometer tests were used to measure the torso muscle endurance, speed capability, and static strength of fingers, respectively; in all tests, speed climbers achieved significantly higher values than recreational climbers (10). Compared to novices, elite speed climbers have also been characterised by a higher absolute and relative strength in upper limbs and higher power in lower limbs (11). Although this characterisation was only based on the evaluation of a few athletes, another study also found that speed climbers have a larger greater tight circumference compared to lead climbers (12). The same authors also reported that speed climbers were taller and characterised by a significantly higher shoulder width (the distance between acromion landmarks) and that the somatic type of speed climbers seems different from lead climbers, but very similar to boulderers (12).

Speed climbers were reported to have a higher body fat mass (13, 14), and their body mass was also found to be significantly higher (15). In men, lean body mass and body height showed the highest correlation with race time (*R*^2 ^= 0.40, *R*^2 ^= 0.70) (16). In eight male recreational climbers, race time also correlated slightly with body fat percentage, and moderately with the ape index and highly (negatively) with arm span (17). Race time also had an insignificant but strong relation to BMI, ponderal index and explosive strength for men (12). Elite female climbers had a lower BMI, ponderal index, and a lower ratio of power to lean body mass compared to male climbers, but none of these differences was significant (16). In a study on five female elite speed climbers (13), some anthropometric variables showed a high significant correlation with female speed climbers' race time. These variables were body height (*R*^2 ^= 0.89), weight (*R*^2 ^= 0.93), and lean body mass (*R*^2 ^= 0.96). In another study, similar findings have been reported: Female climbers' BMI and ponderal index were significantly correlated (*R*^2 ^= 0.52, *R*^2 ^= 0.61 respectively) with the best time of female speed climbers (16). Race time also had an insignificant but strong relation to anaerobic relative power indicators of lower limbs for women. Therefore, it has been speculated that female speed climbers should have a small body size and a high level of explosive power in the lower limbs to succeed in speed climbing (13). However, it should not be neglected that taller athletes could do a countermovement at the start. Larger anthropometric metrics come along with larger angular ranges of motion. Exploiting the entire angular range of motion would result in longer contact times rather than in higher body acceleration. However, a larger range of motion may help to tolerate small movement variation better. Small climbers use holds that cause a greater distance compared to tall climbers, 83% of whom choose holds with a shorter distance (18).

### 4.2. Physiology

In maximal muscle actions lasting up to 5s, phosphocreatine serves as the primary donor for ATP (Adenosine Triphosphate) formation (19). If the motor task continues for more than 5s, anaerobic glycolysis becomes more critical (20). Therefore, the ATP synthesis of elite-level speed climbers is mainly ensured by phosphagen systems and in small quantities by anaerobic glycolysis (21). For other speed climbers and female climbers who are slower, the glycolysis system is used more to provide energy and generate high blood lactate levels, probably resulting in relatively slower muscle contractions and increased climb times. Therefore, slower climbers must improve both anaerobic glycolic and ATP-CP energy systems (22).

It has been postulated that a high power in lower limb muscles relative to lean body mass is needed to be successful in speed climbing (23). The mean of this lower limb power was found to be around 25–26 (W/kg) for six male athletes in the top ten of a speed climbing World Cup in 2016 (21). It has been reported that speed climbers achieved a higher anaerobic power and strength of the lower limbs than lead climbers while lead climbers achieved a higher anaerobic power and strength in the upper limbs (differences summarised in Table 2); however, these differences were not classified as statistically significant (23). Maximum force was also not found to significantly differ between speed, lead, and boulder climbers (15). However, boulderers demonstrated higher maximum power in the pull-up test and develop more upper limb force and maintain it at a high level of velocity compared to lead and speed climbers (15). Furthermore, speed climbers are more advanced in explosive power and speed endurance (23). According to a purely theoretical study, it seems that for speed climbers, the speed component dominates the relationship of both speed-power and speed-endurance while for combined climbers power dominates these relationships (24).

**Table 2:** Summary of made comparisons. Based on the literature review, characteristics of male elite speed climbers were related to other climbers.

Male elite speed climbers compared to	Characteristics
Recreational climbers	Body weight: higher; body fat: lower
	Torso muscle endurance, speed capability test (standing board jump), maximal anaerobic work, upper limbs strength: higher
Novice speed climbers	Absolute and relative strength of upper limbs: higher
	Power of lower limbs: higher
Lead climbers	Height, body mass, shoulder-length and tight circumference, body fat mass: higher
	Anaerobic power and strength of lower limbs: higher
	Anaerobic power and strength of upper limbs: lower
	Aerobic power and muscle endurance: lower
	Coordination: lower
Boulderers	Produce and maintenance external Force at high velocity: lower
	Maximal power of upper limbs (in pull-ups): lower
	Explosive power of lower limbs: higher
Female speed climbers	Body mass index, ponderal index, ratio of power to lean body mass: no sign. differences
	Body height, weight, and lean body mass: no correlation with the male race time
	Power of lower limbs: high correlation with the male race time
	Body height, weight, and lean body mass: high correlation with the female race time

When athletes were asked to jump as high as possible from a sitting position, athletes who were specialised in speed climbing jumped significantly higher than the lead climbers and mountaineers. In the same study, speed climbers were also able to solve pull ups (15 repetitions) and a raise of legs to breast task (20 repetitions) significantly quicker than the other groups investigated (23). In contrast, lead climbers demonstrated a significantly higher aerobic power and muscle endurance than speed climbers by hanging on a 1 cm depth rung, performing the Conconi test (25), and in another pull-up test, respectively (14).

Ozimek et al. (2018) have suggested applying the Margaria–Kalamen formula to estimate the level of sport-specific power of climbers' lower limbs. The formula is based on a test that requests athletes to run up nine steps of a stair after a 6 m sprint to the stairs. Power is then calculated by body mass times gravity times vertical height between the third and ninth step divided by time between stepping on the third and ninth step (21). The extent to which this procedure correlates with performance in speed climbing has not yet been investigated in detail. Another approach, the Wingate test on a bicycle ergometer, has not been shown to correlate with times achieved in speed climbing (26). In contrast, Krawczyk et al. have demonstrated in several studies that an assessment based on a countermovement jump strongly correlates with time in speed climbing (13, 16, 27–29). The explosive power of the lower limbs can also easily be evaluated by a standing long jump, also called Broad Jump. Indeed, this test was able to distinguish professional climbers from recreational climbers (10). Speed climbing training is designed accordingly, as emerged from the interview with the former world record holder: Plyometric exercises for the upper and lower limbs are prioritised, complemented by uphill sprinting, hyper-gravity climbing and one-handed ladder climbs. Reaction time and balance are also trained, but less extensively.

In addition to the blood lactate and power of climbers, the heart rate of seven athletes was investigated during speed climbing. After climbing the 15 m wall, heart rate increases. The rate of change in heart rate (HR acceleration) was found to be on average 2.5 bpm/s with a peak average acceleration of 4.2 bpm/s (30). Also, intense exercise in elite climbers resulted in higher increases in heart rate. Compared to untrained men, who perform very light, non-exhaustive exercises, elite climbers demonstrated a lower heart rate acceleration. Fuss et al. (30) also concluded that five minutes of rest between two speed climbing attempts are sufficient for heart rate recovery. Another study showed that heart rate remains constant during climbing until the climber reaches the top, followed by an immediate increase in heart rate. Then it remained at the peak rate for 3 s; after 59 s, heart rate recovered to the start heart rate (31).

Practically, training programs should prioritise improving explosive power of the lower limbs, jump, relative strength upper limb, speed-endurance (32). In our experience, speed climbers should work on cardiovascular preparation to recover faster next to the plyometric training for lower limbs. However, muscular power is not the only essential factor for success in speed climbing (28). Technical skills, i.e., motor control, substantially affect the power generated and thus, impact the movement speed (21).

### 4.3. Motor control

Speed climbing begins with the start position and ends when the time sensor is touched. The type and sequence of the climbers' movements are crucial for success. Quantitative and qualitative analyses of speed climbing competitions have revealed that the speed on the route depends on how the climber interacts with the holds and how the climber sets the feet and realises a push-off (33). In fact, tactical decisions are central to training in the competitive season (34). To support the learning of these technical aspects of speed climbing, pedagogical observation has been emphasised, among others, to visually characterise the structure and content of both the training process and the competition activity (31, 33). Accordingly, the first author analysed the television footage of the Olympic Games finals in speed climbing to characterise the hand or foot sequence for both women and men. We found that in speed climbing, two limbs interact with the wall more often than three limbs. The results of another study on body position in speed climbing competition in which the two-point position (56%) of the body was more often observed than a triple-point (30%) or a single point position (10%) (33).

**Figure 2:**
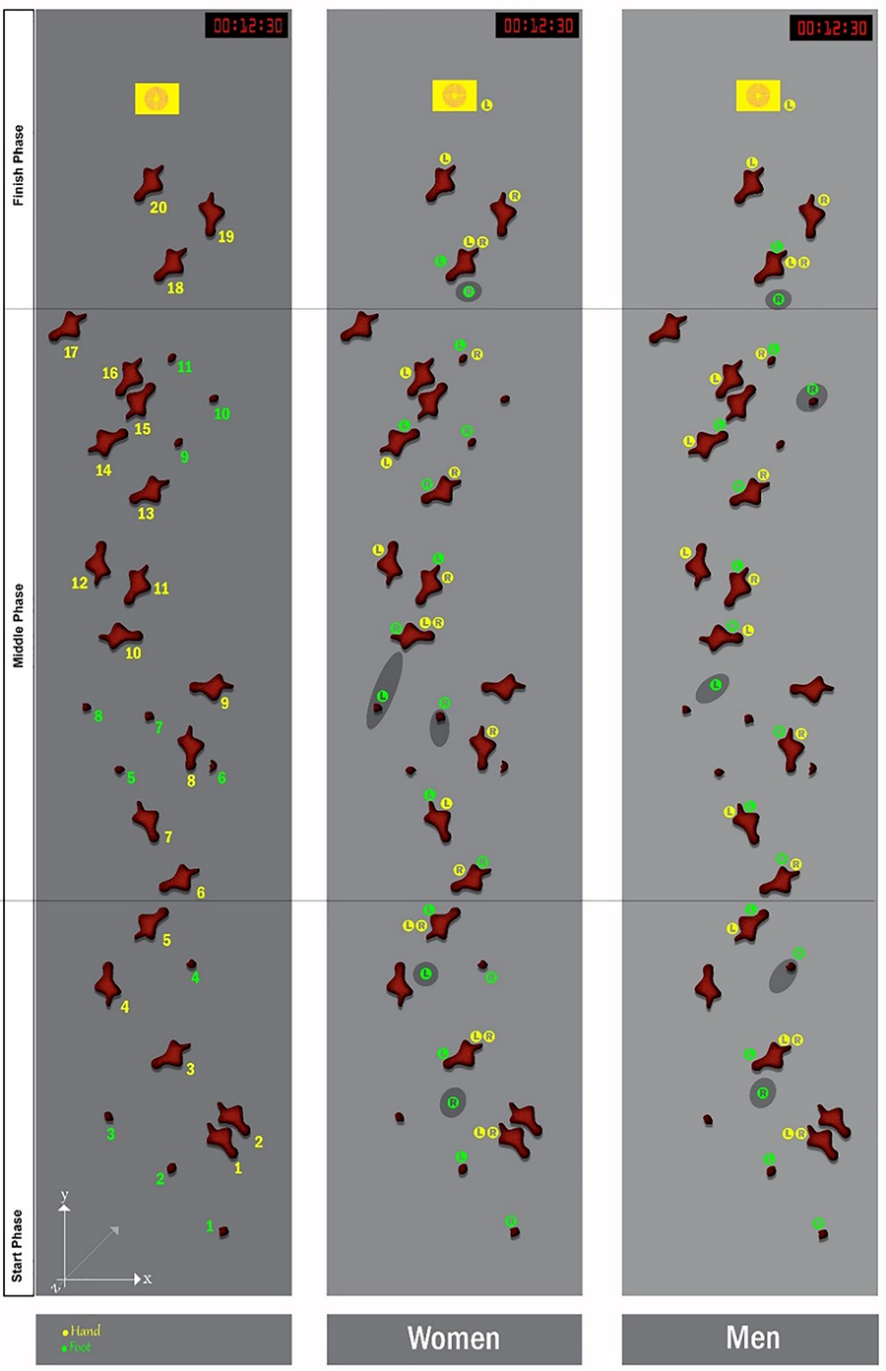
Route setting in speed climbing. Left: Handholds (yellow) and footholds (green) are labelled consecutively. Middle: Typical placement of hands (yellow) and feet (green) during ascent of women at the Olympic Games 2020. Shadow parts represent placement of foot on the surface of the wall. Right: Typical sequence of men.

To provide a more detailed discussion of motor control and related aspects in speed climbing, the route was divided into a start phase, a middle phase and a finish phase, and large holds and footholds were numbered separately to elaborate technical characteristics and to relate them to the literature (see Figure 2).

**Figure 3:**
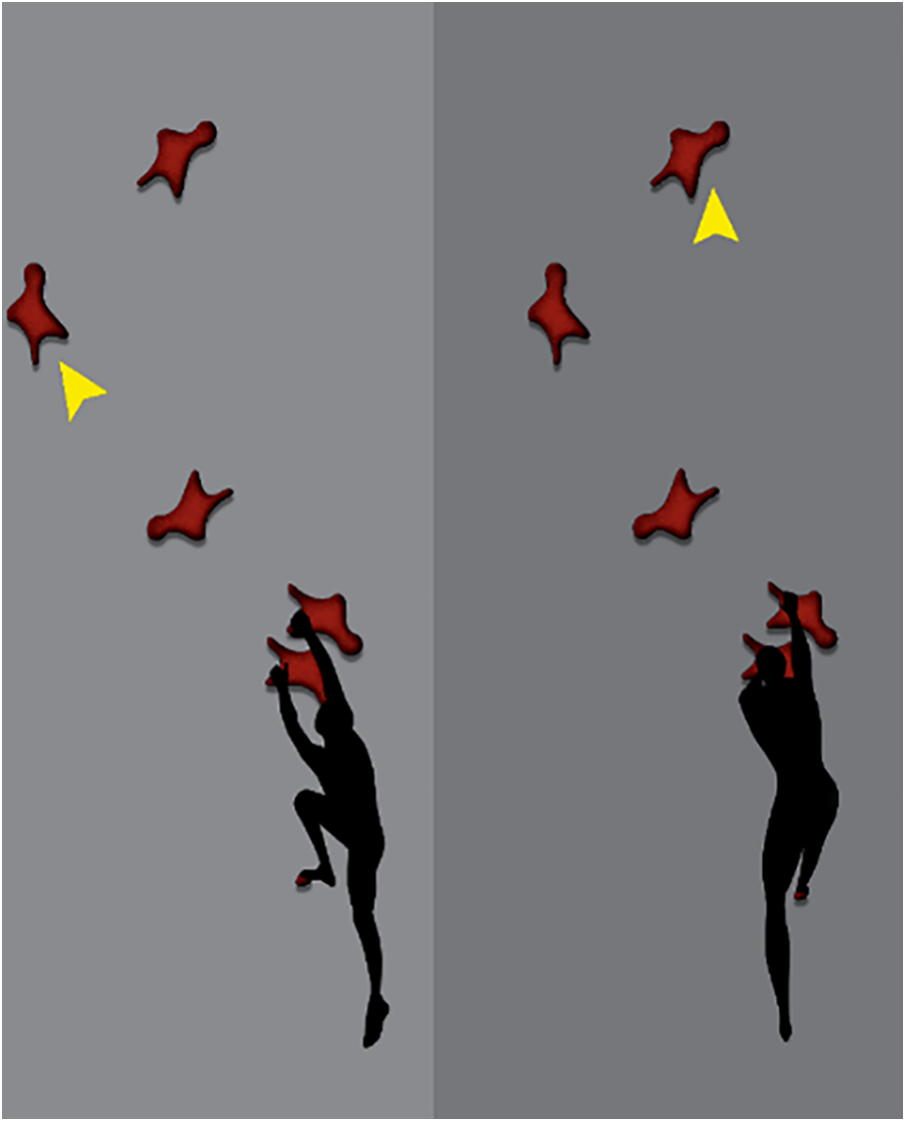
Pose of the classic start (left) and pose of the Tomoa start (right).

**Start phase:** In sprint sports, a successful start is essential and decisive for the athlete's final time. The start position in speed climbing is the only one in which the earth's surface can be used to build up acceleration against gravity. For the authors, the start phase includes the first five handholds (as suggested by others, e.g., 35), even if others (e.g., 31) have also considered handhold 6 as part of the start phase. The posture and placement of the limbs in the start position depend on whether the athlete wants to use handhold 4 or not. The consideration of handhold 4 has changed over the last few years. Initially, handhold 4 was targeted by the athletes: Consequently, they grasped handholds 1 and 2 or just handhold 1 (depending on body size), and oriented their body oblique to the wall, placing the outer part of the right foot on the first foothold. This positioning allows a first acceleration of the body in the direction of handhold 4, which is placed much further to the left compared to the first two handholds (see Figure 3A). In an analysis of IFSC competitions from 2018 to 2019, out of 36 starts analysed, all athletes taller than 1.73 m and about a fifth of shorter ones used handhold 2 with their right hand, while all others placed both hands on handhold 1 (18).

Another grip sequence in the starting phase was named after the Japanese climber Tomoa Narazaki who used the so-called Tomoa skip for the first time in 2018. Here, the start is more frontal to the wall and the right toes instead of the outer part are placed on the first foothold (see Figure 3B). Thereafter, a step-up dyno is performed from handhold 3 to handhold 5 whereby the right foot is placed on the wall and the left on handhold 3. Handholds 2 and 4 are omitted and consequently, handhold 5 is approached in shorter time. In this way, the extent of the lateral movement of the body's centre of gravity (caused by grasping handhold 4) is also significantly reduced, making the vertical movement more fluent. Based on our video analysis, 92% of all athletes started with the Tomoa skip at the Olympic finals in Tokyo.

A study measuring for the first time the interaction forces between holds and eight athletes for the start phase in speed climbing revealed that the more experienced the climber, the more the directions of forces at the hands and feet were aligned (35). This increases the total force and thus also the acceleration.

If the right leg is to contribute to the acceleration of the body during step-up dyno, there must be enough friction between the right foot and the wall to avoid slipping. Accordingly, there must be a wall texture defined by the IFSC that is checked before each competition so that times achieved on different walls remain comparable.

Our analysis of the television footage of the Olympic Games finals in speed climbing also revealed that female athletes touched handhold 4 with the left hand when jumping to handhold 5 to balance their movement away from the wall. The movement perpendicular to the wall seems greater compared to the male athletes because the female athletes are smaller and therefore, they can use handhold 2 less for vertical acceleration. Instead, female athletes accelerated more perpendicular to the wall.

**Middle phase**: We defined this phase ranging from handhold 6 to handhold 18. In this phase, climbers usually climb in different ways, while the style in the first and third phase is quite similar across athletes. Next to the thirteen handholds, seven footholds are present in this phase. Over the last few years, climbers have used fewer and fewer holds, so that by 2021 the elite climbers used a maximum of ten handholds and four footholds to climb straightforwardly to reduce the climbing time. Climbers with a small body size move their legs more frequently. Indeed, a study of 2014 revealed that body size correlated negatively with the number of leg movements while no correlation was found between body size and number of arm movements (36). The same study indicated that a large body size does not give a significant advantage to speed climbers taking into account the entire route. Thus, body size only determines how the leg movement looks like, but not the overall time (36).

**Finish phase:** This phase begins with a dyno from handhold 18 to handholds 19 and 20, which are commonly grasped simultaneously. Thereafter, almost all climbers place the outside edge of the right foot (lateral edge of foot) on the wall, turn their body to the left and place the left foot on handhold 18 to jump again to touch the time sensor with the left hand. It is the final part and essential to do one explosive and accurate movement. It was found that about one fifth of the athletes shorter than 1.73 m touched the sensor with their right hand (18).

### 4.4. Insights from the video analysis of the Olympics 2020

**Hand movements**: At the Tokyo Olympics, the female athletes generally placed their hands and feet in the same way, while the men were found to have different movement sequences for their hands. In particular, we observed that male athletes climbed differently from handhold 11 to handhold 18. Sometimes, handhold 11 was grasped with both hands and then handhold 12 was omitted. Some male climbers used handhold 15 instead of handhold 16 which was subsequently omitted. In contrast to female athletes, men did not place a foot on handhold 4. We also observed that female athletes paired their hands on one hold more often than male athletes (see Table 3). According to a recent study, pairing movements account for 60% of all women's movements during speed climbing (31). In contrast, men commit hand movements in pairs and alternately to a more or less equal extent. In the same paper, it was also stated that climbers who use more handholds on the direct route to the target pad are faster than climbers who prefer fewer handholds and thus execute more high dynos. Higher dynos result in greater fluctuations in vertical speed and also result in undesired body movements perpendicular to the wall. Interestingly, the foothold 11 has also been used as a handhold to prevent the lateral displacement of the body.

**Table 3:** Movement characteristics of speed climbing runs from the quarter finals at Olympic games in Tokyo. A range (min-max) is reported if not all eight runs have resulted in the same number. The total sum of all hand and foot holds is 20 and 11, respectively.

Features	Female climbers	Male climbers
Number of handholds used	15	14
Both hands on one hold (pairing)	5	3–4 (min-max)
Handholds not touched by hands or feet	4	4
Number of footholds used	7	5
Foot placement on wall (friction)	4–8 (min-max)	2–5 (min-max)
Placement of outer/lateral edge of shoe	2–7 (min-max)	0–3 (min-max)

**Foot movements**: At the Olympic Games in Tokyo, female athletes exploited wall friction more often in speed climbing than their male counterparts (see Table 2). We had the impression that at foothold 7 and foothold 8, especially women placed their foot next to the hold because of carelessness (see Figure 2 and its shadow for details where feet were placed).

It should be noted that a more frequent use of the wall surface increases the likelihood of slipping. Indeed, at the Olympic Games, female climbers carried out several consecutive irregular and inefficient foot movements caused by multiple slips that should be omitted during speed climbing. In the first dyno (in the middle phase, from handhold 9 to handhold 10), females used the wall's surface and slipped up to approximately three times to finally put their feet on the footholds. This carelessness in foot movements in the first dyno can be due to the short stature of women. Climbers who have a better personal best time are more accurate in their foot movements and use more footholds. In fact, we have found that at the Olympic Games, slower times were most often due to foot slipping or inappropriate foot movements/placements.

Our analysis also revealed that the female climbers used the outside edge of the foot more often than the male climbers (see Table 3) maybe due to their smaller body size resulting in a greater distance between holds relative to their body size. At the Olympic Games, the average body size was 1.63 m for women and 1.77 m for men. If this difference in body size were taken into account in the length of the route, it would only be 13.88 m long for the women instead of 15 m. Relative to their body size and related to their centre of mass, women must jump higher in all dynos to reach the targeted hold or the time sensor. In other words, the path of their centre of mass is longer at the end of the route (37). That female speed climbers generally have to jump higher than male climbers may explain our observation why women use the lateral part of their feet combined with hip rotation or lateral shifting of the hips more often than male speed climbers: this movement might be time-consuming, but it helps to reach the next hold. It is possible that climbers do not use the lateral part of their feet but just turn their hip and laterally displace their centre of mass above the support of the foot to be able to push themselves longer. Another video analysis study stated that during the race, men push themselves with their feet much more often off holds (13 times) than off the wall (4 times). The average frequency of females' hand movements was calculated at 2.53 movements/s, which is 11% less than in men (2.8 movements/s). In the movement of the legs, the frequency is 2.5 movements/s for women and 2.9 movements/s for men. These differences were attributed to the different physiological conditions of women and men (9).

**Movement errors**: When analysing the Olympic Games, it was found that movement errors, e.g., slipping of hand or foot on a hold or wall, occur more often in the middle phase (see Figure 2), which is consistent with the results of another study (31). We have also noticed that more errors were made in the women's final than in the men's final (seven vs. five). Female athletes failed most frequently at the first dyno or lost significant time there. In a former analysis of the 2019 World Cups, women made errors mainly in the last phase (38). Male athletes slipped especially with their feet when they placed them against the wall to use the friction on it (see Table 3). Previous studies have also shown that errors in foot placements are quite common: In the 2007 Speed Climbing World Cup, most movement errors happened during foot placement (56.3%), followed by errors in grasping (31%) (33). Given the error rate - studies showed that many climbers make crucial mistakes in the final round of competitions (9, 38) - it is understandable that technical training has become more important (9). It is recommended that climbers try to use footholds that are closer to the direct route rather than relying on the friction of the wall surface (the more the wall surface is used, the more likely slipping is). For beginners, however, the focus is first on learning the movement sequence; only later faulty foot movements or false starts are more prevalent, as our interviews with experts revealed.

In general, the following suggestions should be considered in order to optimise speed climbing performance in terms of movement sequences. The Tomoa start could be the best start for men but not for women (the female world champion of 2022 started quite individually). Females could touch handhold 4 only by the left hand. Men should avoid rotating the hip resulting in usage of the lateral side of the foot. More ladder and alternating hand movements, less pairing of two hands, jumping less high, and using more grips that are on the direct line upwards improve one's personal best time, too. Particularly in the middle phase, training should focus on using the foot holds instead of relying on friction between wall and shoe. According to our interviews, women should climb according to their anthropometry and not try to imitate men's movements.

### 4.4. Biomechanical metrics characterising movement quality

From a biomechanical perspective, various kinematic and kinetic metrics can characterise performance in speed climbing. Essential is the trajectory of the centre of mass (COM): how directly and fluently does the climber approach the target pad? Many velocity changes or jerky movements of the centre of mass are disadvantageous.

**Fluency**: To describe the extent of a fluent climbing movement, or rather the opposite, the metric “entropy” was used for the first time by Cordier et al. in 1993 (39, 40). Entropy refers to a thermodynamic concept that indicates the degree of disorganisation of a system. In climbing, entropy describes the disorganisation of the climber's COM path. Accordingly, minimum entropy is aimed for (40). Ideally, climbers would climb on a direct path, which is a straight line to the target pad. Any deviation from this direct line will lengthen the path and thus also the energy expenditure and the time required. However, the holds of the route do not allow a direct ascent. A specific geometric entropy can then be assigned to each chosen ascent. This entropy H is calculated under consideration of the path covered by the pelvis L and the circumference of the convex hull C around this path as follows: H = log_n_(2L/C) (41). The entropy is minimised when the pelvis moves closer to the direct line and lateral movements are reduced (37, 42, 43). Cordier et al. (1994) were able to show that this geometric entropy can also be used to assess the skill level: The more experienced the climbers, the smaller the entropy (42). In speed climbing, minimum entropies were determined in the order of 0.10 and 0.14 for men and women, respectively (37). Even if geometric entropy constitutes a practicable measure of fluency, it considers purely spatial quantities in the plane of the wall. Movements perpendicular to the wall and temporal aspects such as speed variations, whereby rest positions in bouldering or lead represent an extreme variant, are not taken into account (44).

To describe both spatial and temporal aspects of fluency, the jerk of an ascent was considered. The jerk results from the third time derivative of the position and describes the change in acceleration. In climbing, expertise and route design influence the total jerk of a route: the more experienced a climber is in a particular route and thus, the more fluently the climber moves upwards, the smaller the total jerk (44). The jerk is also small if a route can be mastered by ladder movements, i.e., holds can be grasped from above and hip rotations are not required. The route in speed climbing, i.e., its holds, allows laddering, so that in comparison to the other climbing disciplines—if such a comparison is possible at all—entropy and jerk of the COM are smaller in speed climbing. The trajectory of a marker attached to the climber's harness has often been monitored to assess fluency. The marker on the harness was assumed to be representative of the trajectory of the body's COM. This assumption is only true to a certain extent: By recording the body segments of one athlete in detail, vertical deviations between simplified and detailed COM trajectories of up to 24 cm were found in speed climbing. These deviations become obvious, for instance, when the pelvis rests and the legs climb even higher (45). In order to assess the performance in speed climbing, it is perhaps even more suitable to look at the velocity of the COM, since climbers ascend continuously and as fast as possible. Male and female elite climbers have average vertical velocities of 2.5 m/s and 2 m/s respectively (26).

**Velocity of COM**: During the ascent, the velocity of the COM varies: In the start phase, the vertical velocity increases abruptly, then it decreases again, with the lowest vertical velocities occurring after the dynos and at the end of the phase of a classical start (see Table 4). Speed reductions are more pronounced in women than in men (37). Fluctuations in velocity result in the variations in acceleration and jerk, so climbers try to keep their vertical velocity constant and minimise the deceleration and jerk. Climbers also try to minimise lateral movements and movements perpendicular to the wall and accordingly also keep these velocity components small. Especially in the two dynos (from handhold 8 to handhold 9 and from handhold 16 to handhold 17), the vertical velocity decreases with the grab at the end of the dyno while during the dyno the velocity perpendicular to the wall increases significantly, which results from the swing movement. The lateral velocity component increases while climbers move up from handhold 7 to handhold 8 and from handhold 13 to handhold 14. The topology of holds results in a decrease in vertical velocity (46). In a study on three speed climbers, it was also found that vertical velocity correlated negatively with contact time (the faster, the shorter the contacts) and positively with contact forces (the faster, the higher the contact force) (47).

**Table 4:** Times and average vertical velocities in different phases at Olympic games in Tokyo for male and female athletes.

		Time (in s)				Average vertical velocity (in m/s)			
		Start	Middle phase		Finish	Start	Middle phase		Finish
		Start—handhold 5	Handhold 5—11	Handhold 11—18	Handhold 18—top	Start—handhold 5	Handhold 5—11	Handhold 11—18	Handhold 18—top
Male	Mean	0.520	0.453	0.494	0.446	1.68	1.92	2.07	1.86
	Max	1.070	0.706	0.786	0.762	2.24	2.43	2.70	2.44
	Min	0.390	0.360	0.377	0.340	0.81	1.23	1.29	1.09
Female	Mean	0.590	0.587	0.891	0.550	1.48	1.49	1.71	1.50
	Max	1.103	0.886	0.820	0.733	1.93	1.89	2.14	1.91
	Min	0.453	0.460	0.476	0.435	0.79	0.89	1.24	1.13

Climbers can increase their vertical velocity and accelerate their movement in the vertical direction in two sections characterised by a topology of holds enabling laddering (37). These two sections are before and after the first dyno of the speed route. Thus, climbers must strive at the last hold of the first dyno to increase the vertical acceleration. Accordingly, the average speed is the highest in the middle phase (see Table 4).

**Figure 4:**
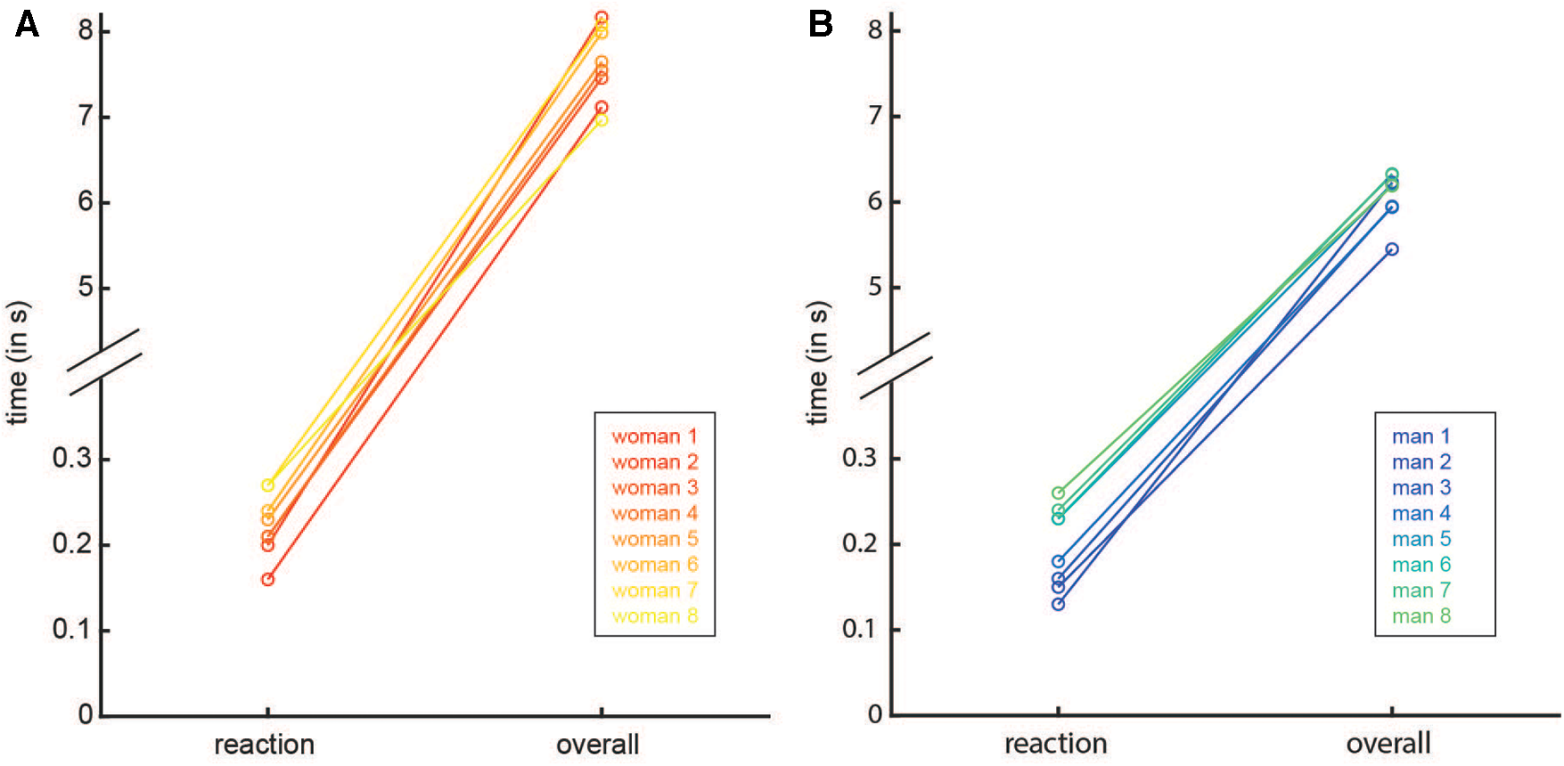
Reaction time vs. the overall time of the eight individual best runs for women (**A**) and men (**B**) at the Olympic games.

**Reaction time:** The time between the occurrence of a stimulus, i.e., start signal, and leaving the start pad is defined as reaction time. Speed climbers have achieved the highest “functional mobility of nervous process” among boulders, lead climbers, and mountaineers (48). The kind of training and discipline require total concentration in maximal force effort (48). At the Tokyo Olympics, the mean reaction time for men was 0.238s and for women 0.246s in the 1/4-final and final rounds (see Figure 4), with no statistically significant difference between the two genders. There was no correlation between reaction time and final time which is in agreement with former work (38).

**Force**: The musculature of the lower extremities is the essential “motor” in speed climbing (14). However, the force development in the upper extremities was investigated under various aspects, too. One study on three speed climbers reported that the higher the velocity of the climber was, the higher the contact forces at the hold were, and the shorter contact time was. Thereby, the relationship between velocity and contact forces was not linear: at higher velocities, the contact forces increased to a lesser extent. The magnitude of the finger reaction force has also been found to correlate with skill (47). A test in which a pull-up had to be performed as fast as possible under various additional loads showed that speed climbers achieved lower speed and power (product of force times speed) than boulderers. Boulderers showed a higher ability to generate force at high speed (15). These differences in arm power are not surprising since in speed climbing leg power is more essential for propulsion. Another study concluded that the cycle ergometer test has a low practical value for predicting record time (26). Other studies on the relation of force and velocity stated that speed and force are inversely related to each other, and this relation is hyperbolic in speed climbing (24, 49). The decreasing impact of the speed-force factor is accompanied by an increase in the importance of other factors, probably coordination of limbs, in achieving high sports results (50).

**Balance:** It seems that balance has become more critical in climbing because of using different volumes and slope holds in route settings. Balance is defined as an ability to maintain the line of gravity of mass in the base of support (51). A few studies have investigated the balance skills of climbers, but only one of them investigated the relation of different assessments of balance to the performance in the three disciplines of sport climbing. It was found that the outcome of the flamingo test significantly correlated with the results achieved in bouldering and lead climbing whereas the outcome of the crosswise standing on a balance bench test significantly correlated with the results achieved in speed climbing. The latter correlation was explained by the fact the foot position of this balance test corresponded to the mostly present foot position in speed climbing, namely perpendicular to the wall (52). However, these correlations should be interpreted with caution, as the athletes studied demonstrated much higher skills in bouldering and lead climbing than in speed climbing.

**Coordination:** Coordination is defined as a spatio-temporal sequence (where and when) of movements of different parts of the body. Different kinds of coordination can be considered, i.e., intra-muscular coordination, inter-muscular coordination, musculoskeletal coordination, and inter-articular coordination (42). Some studies suggest that coordination can be considered as a crucial factor in speed climbing along with other, performance-determining factors such as strength, technique, speed component, psychology and anthropometrics (21, 26, 50). Coordination of climbers has been assessed by several tests: The “plate tapping” test was used to distinguish elite speed climbers from recreational climbers, however, no significant group difference was found (10). In contrast, a double–plate postughraph has been proposed to reasonably assess coordination skills in climbing (53). Athletes of the three different sport climbing disciplines have also been monitored by other coordination tests, i.e., the obstacle course backward in seconds, coordination with a baton, and 20 steps with a baton. Results revealed that athletes with better coordination achieve better results in lead climbing. No such significant correlation was found for success in bouldering or speed climbing for the success in bouldering or speed climbing no such significant relation was observed (54).

## 5. Summary

This paper attempted to address the factors influencing success in speed climbing. Studies on physiology have shown that elite speed climbers are characterised by high anaerobic relative strength, endurance and great explosive strength of the lower limbs. The phosphagen system dominates energy provision in elite male speed climbers; they need to train accordingly. Slower speed climbers, on the other hand, have to improve their anaerobic glycogen system. The amount of lactate in the blood correlates directly with the climbing time. Performance in speed climbing correlates with performance in jump tests (squat and countermovement) and standing long jump tests.

With regard to the movement technique, the placement of the feet seems to be elementary in order to optimally use the strength of the legs and to avoid slipping off the wall. In the Tomoa start method, women should provisionally touch handhold 4 to avoid a reduction in vertical velocity and to keep the centre of mass close to the wall. In general, speed climbers should try to climb in a direct line from the start to the top and avoid lateral movements. Highest vertical velocities were found between handholds 11 to 18 after the first dyno whereas in the final phase is the slowest phase. Long dyno movements seem to result in a loss of vertical velocity unless.

Compared to lead climbers, male elite speed climbers are taller, heavier, and their body fat mass as well as anaerobic power and strength of the lower limbs are greater. Speed climbers are weaker than lead climbers in terms of anaerobic power and strength of the upper limbs, aerobic power and muscle endurance. Compared to boulderers, male elite speed climbers achieve a lower rate in producing and maintaining force at high movement velocities and maximal power in the upper limb pull-up test. In contrast, the explosive lower limb power is higher in speed climbers than in boulderers. Elite female speed climbers had a lower BMI, ponderal index, and a lower ratio of power to lean body mass compared to male climbers.

## 6. Practical suggestion

Even though not all speed climbers will reach an elite level, aspects of this review can help to improve personal best times. In the interviews, the coaches mentioned that in speed climbing, due to the specific motor skills required and the standardised climbing route, one can develop from a beginner to an established athlete more quickly than in other climbing disciplines, even though achieving a very good time is then very training-intensive. The following general practical advice emerged:

• Women should climb according to their body dimensions and should not try to imitate men's movements.
• While men may focus on ladder movements, women may train pairing their hands on a hold.
• Beginners should avoid movement mistakes and train coordination while elite climbers should avoid starting errors and wrong foot movements.

## 7. Limitations

This paper summarises the literature on factors related to climbing performance in speed climbing. Some papers are only case studies; however, these have been included in this paper due to the paucity of scientific literature. As a result, some claims about performance-related factors do not seem to be widely supported, but we have pointed this out in the discussion or emphasised the consistency with other study results. Most biomechanical and physiological papers considered were descriptive and did not include statistical analysis, making it difficult to compare results and formulate generalisable conclusions.

## 8. Future research

Future studies should focus even more on the physiology and biomechanics of speed climbing, especially to highlight the differences between men and women and between different climbing strategies. It will be beneficial to recruit a reasonable number of participant and to study the different sections of the route separately. The results of such studies will help to optimise training measures and improve climbing times.
