# Supplementary material for: Performance indicators in speed climbing: insights from the literature supplemented by a video analysis and expert interviews
Source: Front Sports Act Living. 2023 Dec 22;5:1304403. doi: 10.3389/fspor.2023.1304403 (PMC10766694; doi:10.3389/fspor.2023.1304403)
Supplement: Supplementary file 1 [file Table1.docx]

Appendix Table 1: Summary of articles included in the systematic review.

| **Domain of study** | **First Author, year, country (# reference)** | **Title of publication** | **Participants** | **Type of analysis** | **Metrics evaluated** | **Main results** |
| --- | --- | --- | --- | --- | --- | --- |
| **Anthropo-metrics** | Ryepko O.A., 2013, Ukraine (12) | Morphological characteristics of elite athletes, specializing in speed climbing, climbing and alpinism | 10 speed climbers, 10 difficulty climbers, 6 alpinists  (all world-class, male, 19-22 years) | Comparative analysis | Body length, body mass, lengths and masses of body segments, circumferential sizes of body segments | Speed climbers’ body length > difficulty climbers  Speed climbers’ thigh circumference > difficulty climbers  Speed climbers’ shoulder width was the greatest |
| **Physiology** | Fuss F.K., 2020, Austria (30) | Heart rate behavior in speed climbing | 5 females,  2 males  (near-elite speed climbers) | Descriptive | Heart rate on 10- and 15m walls | 10m wall: mean peak heart rate 165bpm  15m wall: mean peak heart rate 176bpm |
|  | Gąsior P., 2020, Poland (53) | Kinesthetic differentiation, kinematic and dynamic parameters in sport climbing competitors of varying ability levels | 30 male climbers:  10 at amateur level  10 intermediates  10 advanced climbers | Comparative analysis | Relative strength, power, climbing speed by accelerometer A two-plate stabile graphic scale test (double-plate posturograph) | Relative strength and relative power of upper limbs as well as climbing speed significant determinants of the skill level. No statistically significant differences in kinesthetic movement differentiation abilities in lower limb measurements. |
|  | Guo F., 2018, China, (22) | Changes in blood lactate and muscle activation in elite rock climbers during 15m speed climb | 6 females,  6 males  (national elite) | Correlation | Muscle activity, blood lactate, climbing time | Median frequency of upper extremity sEMG decreased more than that of lower extremities.  Positive sign. correlation between blood lactate and climbing time. |
|  | Ignjatović M, 2016, Serbia (52) | Relation and influence of balance on the result in sport climbing | 11 female participants of Youth World Cup | Correlation | Balance (one leg standing along on a balance bench as well as crosswise standing on a balance bench), climbing time | Balance abilities correlated with success in bouldering but not with success in lead and speed climbing (significance for speed climbing was on the borderline). |
|  | Kozina Z., 2014, Ukraine (24) | Theoretical-methodological study of development of power-speed in climbing | 10 speed climbers, 10 difficulty climbers, 6 alpinists  (all world-class, male, 19-22 years) | Theoretical analysis | Speed- force readiness (strength, speed and speed-strength, endurance) | Speed-force readiness (strength, speed and speed-strength, endurance) has an inverse, hyperbolic relationship. |
|  | Kozina Z., 2015, Ukraine (48) | Psychophysiological possibility of mountaineers and climbers specializing in speed climbing and climbing difficulty | 10 speed climbers, 10 difficulty climbers, 6 alpinists (all world-class, male, 19-22 years) | Comparative analysis | Psycho-physiological methods of research (speed of plain and complex responses, determination of strength and mobility of nervous system) | Speed climbers’ mobility of the nervous system > alpinists Training and competition of rock climbers require concentration in conditions of maximal or close to maximal force efforts. |
|  | Krawczyk M., 2021, Poland (26) | Predicting performance in speed climbing: accuracy of the force-velocity test on a cycle ergometer | 10 junior male speed climbers | Correlation | Force-velocity characteristics of lower limbs measured by a cycle ergometer, personal best in speed climbing | Cycle ergometer test of low value to predict speed climbing performance. |
|  | Krawczyk M., 2015, Poland (11) | Value of select displays of strength and speed abilities in speed climbing at the highest sport level - analysis of cases | 4 members of the Polish national speed team | Correlation | Body height, body weight, dynamometrical test of maximum static strength of upper limbs. Maximum anaerobic work and strength and endurance of torso muscles, frequency movement of upper limbs | Speed climbers benefit from a high level of power in lower limbs and should pay attention to relative strength. A standing jump test is a valuable tool for speed climbing. |
|  | Kozina Z., 2016, Ukraine (49) | Mathematical basis for the integral development of strength, speed and endurance in sports with complex manifestation of physical qualities | 10 speed climbers, 10 difficulty climbers, 6 alpinists (all world-class, male, 19-22 years) | Comparative analysis | Speed, strength, endurance, physical qualities | The components of physical readiness (strength, speed and endurance) are inversely related. The dependencies are hyperbolic |
|  | Kozina Z., 2020, Ukraine (32) | Experimental substantiation of the program of the annual cycle of preparation of climbers 16-17 years to the competitive discipline "Climbing Combined" | 23 members of Ukraine youth team | Comparative analysis | Speed climbing performance | Experimental group (specific training program lasting 1 year) improved performance in speed climbing, standing jump, 100m run and hanging time whereas control group showed no significant improvements. |
|  | Levernier G., 2020, France (15) | Force–velocity–power proﬁle in high elite boulder, lead, speed climber competitors | 11 boulderers, 8 lead climbers, 5 speed climbers  (all high elite climbers) | Comparative analysis | 2 pull-ups at different percentages of their body mass (with additional weight) | Boulderers were able to develop higher forces and to maintain higher speeds compared to the other climbers investigated. |
|  | Ryoko O.A., 2013, Ukraine (23) | Features and functionality of speed and power capabilities of elite climbers and various types of rock climbing | 10 speed climbers, 10 difficulty climbers, 6 alpinists (all world-class, male, 19-22 years) | Comparative analysis | Heart rate, standing high jump, pull-ups to waists, time for 15 chin-ups, hanging on a 1cm rung | Heart rate at rest: alpinists < difficulty climbers < speed climbers  Standing high jump: speed climbers > difficulty climbers > alpinists  Pull-ups to waist: difficulty climbers > speed climbers > alpinists  15 chin-ups: speed climbers < difficulty climbers < alpinists  Hanging: alpinist > difficulty climbers < speed climbers |
|  | Krawczyk M., 2021, Poland (29) | Informativeness of vertical jump attempts with deferring spatial structure in speed climbing | 5 men and 5 women, member of Polish speed national team | Correlation | Counter movement jump with/without arm swing, with rotation around the body axis, motor reaction time task. | Correlation between best time and counter movement jump with rotation around body axis > correlation between best time and counter movement jump without arm swing > correlation between best time and counter movement jump with arm swing |
|  | Ozimek M., 2018, Poland (21) | Evaluation of the level of anaerobic power and its effect on speed climbing performance in elite climbers | 6 male participants of a World Cup in speed climbing | Correlation | Video analysis to determine climbing time, reaction time, and to deduce mechanical power Body height, body weight based on IFSC webpage | Relative power: 24.56W/kg to 27.0W/kg  Only relative power sign. correlated with fastest race time. |
|  | Stanković D., 2017, Serbia (54) | The influence of coordination on the results in sports climbing: the underlying relations | 11 female participants of Youth World Cup | Correlation | Polygon run backwards in seconds, coordination with a baton in seconds, 20 steps with a baton, Points achieved in bouldering, lead, and speed climbing | Polygon run & 20 steps with a baton sign. correlated with points achieved in lead climbing. No other sign. correlation between metrics of coordination and performance in a climbing discipline. |
| **Anthropo-metrics & physiology** | Bolboli L., 2018, Iran (14) | مقایسه نیم رخ های آنتروپومتریکی و فیزیولوژیکی مردان صخره نورد سرعتی و سرطناب تیم ملی ایران. | 10 speed climbers,  10 lead climbers | Comparative analysis | Height, weight, arm span, foot length, body fat percent, circumference of forearm and strength of upper limbs by two kinds of pull-up test, dynamometer, foot press | Anaerobic power and strength of upper and lower limbs: no significant difference between speed and lead climbers.  Aerobic power and muscle endurance: lead climbers > speed climbers |
|  | Krawczyk M., 2014, Poland (10) | Somatic traits and motor skill abilities in top-class professional speed climbers compare to recreational climbers | 5 elite speed climbers,  10 recreational climbers | Comparative analysis | height, body mass, upper limb length, percentage of fat, body mass index, static strength of upper limbs, standing broad jump, sit-ups, sit and reach, spread sit, maximal anaerobic work, envelope run | Speed climbers showed significantly better results in sit-ups, standing broad jump, maximal anaerobic work and envelope run. No other significant differences reported. |
|  | Krawczyk M., 2017, Poland (28) | Level of selected speed ability indexes of lower limb in relation on climbing time in the speed | 5 male elite speed climbers | Correlation | Body height, body weight, lean body mass, percentage of fat tissue, length of the upper limbs, length of the lower limbs and power of lower limb by counter movement jump with/without arm swing, climbing time | Power of the lower limbs showed highest correlation with with climbing time. Other metrics showed strong but insignificant correlations. |
|  | Krawczyk M., 2018, Poland (13) | Anthropometric characteristics and anaerobic power of lower limbs and their relationships with race time in female speed climbers | 5 female elite speed climbers | Correlation | Body height, body weight, fat percentage, lean body mass, ponderal index, body mass index Counter movement jump (to compute maximal anaerobic power) Personal best time | Body height, body weight, and lean body mass sign. correlated with personal best time significant correlations. No other sign. correlations reported. |
|  | Krawczyk M., 2020, Poland (16) | Selected morphofunctional characteristics and their correlations with performance of  female and male speed climbers | 8 females,  10 males  (participants of Polish Speed Climbing Championships) | Correlation,  comparative analysis | Body height, body weight, fat percentage, lean body mass, ponderal index, body mass index Counter movement jump height (to compute maximal anaerobic power) Best time during competition | Sign. differences between female and male competitors in race time, body height, body weight, percentage fat tissue, lean body mass, counter movement jump height (and computed power). In females, body mass index and ponderal index correlated with race time. In males, counter movement jump height (and deduced maximal anaerobic power) sign. correlated with best race time. |
|  | Lau E., 2021, England (17) | Identifying physiological demands of speed climbing within a sample of recreational climbers. | 8 recreational male climbers | Correlation | Body height, body weight, fat percentage Isometric mid-thigh pull test, upper limb power test, countermovement jump Lead and tope-rope climbing skills, speed climbing performance | Speed climbing time had moderate negative correlations with isometric mid-thigh pull test and low positive correlations with body fat percentage. No significant correlation with countermovement jump. High negative correlations were seen for climbing skills. |
|  | Krawczyk M., 2020, Poland (50) | Value of speed capabilities in youth speed climbing at high sports level | 13 female youth 13 male youth  (participants of European Junior Cup) | Correlation,  comparative analysis | body height, body weight, percentage of fat tissue, body mass index,  Wingate test on cycloergometer to estimate anaerobic capacity, maximum muscle power | Lean body mass, total work and peak power were higher in older youth (female and male).  In younger females, race time sign. correlated with relative peak power. No other sign. correlation with race time observed. |
| **Motor control** | Chen R., 2022, China (35) | A time-motion and error analysis of speed Climbing in the 2019 IFSC Speed Climbing World Cup final rounds | Videos of six Speed World Cups in 2019, 384 climbs in total | Correlation | Reaction time, split times, overall time | No correlation between reaction time and overall final time. In women, time of previous phase correlated with next phase. |
|  | Kassirer E., 2021, Canada, (18) | Characterizing route strategy in professional speed climbing with respect to athlete height | 18 elite speed climbers | Descriptive | Height | Climbers greater than 1.73m minimised the number of holds. Conversely, short climbers used more holds and their total path climbed was longer. |
|  | [Krawczyk](https://scholar.google.com/citations?user=TukRP1wAAAAJ&hl=en&oi=sra), M. 2020, Poland  (27) | [The force, velocity, and power of the lower limbs as determinants of speed climbing efficiency](https://www.wbc.poznan.pl/dlibra/publication/584763/edition/497265) | 8 elite speed climbers | Correlation | countermovement  jump, climbing power, Velocity | Velocity, power, the height of the CMJ jump, and the special  climbing power closely correlate with climbing time |
|  | Shulga A.S., 2010, Ukraine (33) | Technique of competition activity of sportsmen, specialized in speed climbing | Youth championships 2009 & Championships 2008 in Ukraine, World Championships 2007 (each time 16 participants) | Descriptive | Movement techniques and errors | Double support (53-56%) more often applied than single or triple support.  Foot placement errors occurred more often than errors while grasping a hold or errors related to movement sequence. |
|  | Shulga O., 2014, Ukraine (37) | Model characteristics of climbers, specializing in climbing at speed | TOP-10 of the  international male ranking in 2013 | Descriptive  (video analysis) | Body height, number of hand and leg movements. | The main discriminant feature is the number of foot movements.  The number of kicks by the legs is the main significant indicator that allows for the distinction between high-skilled climbers. |
|  | Shunko A., 2020, Russia (31) | Competitive modelling in speed climbing | 18 members of the adult and youth men's national team of Russia | Descriptive | Body height, body weight, leg length, heart rate, hand/leg movements Abilities of the cardiovascular system (heart rate monitor) | Women moved hands and legs more often than men. Men demonstrated more alternating hand movements than women whereas women showed more simultaneous hand movements. |
|  | Shunko A., 2020, Russia (9) | Video analysis of competitive activities as a way to control of tactical, technical and physical activities training of qualified rock climbers specializing in speed climbing | 112 runs of female and male speed climbers participating in 7 international competitions in 2019 | Descriptive  (video analysis) | Number of movements with hands (alternately, simultaneously) and feet (in friction, on a hook) | Average number of hand and foot movments in men: 2.8 times /s and 2.9 times / s. In women: 2.5 times / s each.  Women moved their hands more often at the same time than alternately, while men grasped them equally often at the same time or alternately. |
|  | Shunko A., 2021, Russia (34) | Competition model of speed climbers in climbing |  | Descriptive, correlation | Number of movements with hands  Leg length, height, arm span, weight Heart rate | No correlation between anthropometry and number of foot and hand movements. Lower heart rate resulted in lower recovery time. |
|  | Uvarova N., 2020, Ukraine (2) | Research on the results of speed climbing of female climbers on the speed climbing wall between 2011 and 2019 | List of results of competitions from 2011 till 2019 | Descriptive | Women’s record time in Ukraine and in the world | Women's record times have improved over the years and the time differences between the top athletes have become smaller. |
| **Biomechanical metrics** | Fuss FK, 2006, Singapore (47) | Dynamics of speed climbing | 3 climbers | Descriptive | Contact time at the hold, contact forces and shock spike at initial contact Climbing velocity | The higher the climbing speed, the higher the finger reaction forces at the hold, the higher the shock spike at initial contact and the shorter the contact time. |
|  | Legreneur P., 2018, France (42) | Body mass center optimization in sport climbing | ---- | Review | Path of the centre of mass | Author concluded that path of centre of mass and the metrics derived from it such as entropy and mechanical energy reliably indicate speed climbing performance. |
|  | Legreneur P., 2019, France (38) | Kinematic analysis of the speed climbing event at the 2018 Youth Olympic Games | 42 sport climbers (21 men and 21 women) | Descriptive | Kinematics of the pelvis | Performance differences in men and women were explained by their ability to minimize lateral displacements for increasing fluency, to maintain high-velocity levels all along the acceleration phases, and to minimize velocity decreasing after dynos. |
|  | Reveret L, 2018, France (45) | 3D motion analysis of speed climbing performance | Case study | Descriptive | Velocity profile, trajectory of pelvis | Marker attached to the harness has a mean distance of 12.5cm +/- 2.4cm to the true centre of mass of the participant during ascent. |
|  | Reveret L, 2020, France (46) | 3D visualization of body motion in speed climbing | Case study | Descriptive | Trajectory of harness marker | Path of the harness marker deviated on average about 9cm in the plane of the wall from a centre of mass estimated from a 3D mesh of the body registered to a video recording. Out of plane mean deviation was about 24cm. |
|  | Wolf P., 2021, Switzerland (36) | Performance analysis in speed climbing: first insights into accelerating forces during starting phase | 8 elite speed climbers | Descriptive | 3D force | The more experienced the climber, the more the directions of forces at the hands and feet were aligned. |
