# Supplementary material for: Performance indicators in speed climbing: insights from the literature supplemented by a video analysis and expert interviews
Source: Front Sports Act Living. 2023 Dec 22;5:1304403. doi: 10.3389/fspor.2023.1304403 (PMC10766694; doi:10.3389/fspor.2023.1304403)
Supplement: Supplementary file 2 [file Table2.docx]

Appendix Table 2: Summary of interviews

| Question | Consolidated answer |
| --- | --- |
| How can male and female climbers achieve better times? | The focus should be on reaction time and the start, but also on the entire sequence of movements, whereby the placement of the feet is crucial. Women should deliver their own performance and not imitate the movements of male athletes. |
| What are the most prevalent mistakes made by novice and elite speed climbers? | Novice climbers: Sequence of movements and gripping the wrong hold.  Elite climbers. Faulty start and wrong foot movements. |
| Do you think speed climbing athletes can achieve higher skill levels easier, i.e., from novice to elite, than athletes of other climbing disciplines? | Reaching a high national level is possible more quickly than in other disciplines, as the number of speed climbers is generally lower than in bouldering and lead, but breaking the world record is not easy. |
| Do you prefer doing more high dynos with less gripping than short ones catching more holds? | Dynos are not suitable for all speed climbers. Their usefulness depends on body size and the ability to make a powerful jump. |
| Is laddering (i.e., alternate grasping) a better strategy than grasping holds with both hands simultaneously (pairing)? | In general, laddering move is preferable. For smaller women, pairing can help them reach the next hold. |
| Any comments about current practical training methods and exercises for speed climbing athletes | The focus is on plyometric exercises for the upper and lower limbs, supplemented by sprinting uphill, hyper-gravity climbing and one-handed ladder climbs. Reaction time and balance are also trained, albeit less extensively. |
